# Commencement Exercises

**Published:** 1866-03

**Authors:** 


### COMMENCEMENT EXERCISES.

The Commencement of the Ohio College of Dental Surgery took place, in the College on the evening of the 21st of Feb. The lecture room was filled to overflowing; an extempore address was delivered by the President of the Board of Trustees, in which he gave much good counsel and practical advice upon professional matters, especially to those about to enter upon its duties. He then conferred the degree of D. D. S. upon the following named gentlemen, viz. P. J. Collins, W. H. Pabodie, M. Stout, Miss L. B. Hobbs, D. A. Wilder, H. Newington, C. C. Chittenden, W. H. Woodward, A. E. Lyman, Geo. L. Shepard, J. Nelson, R. C. Morgan, G. W. DeCamp, J. P. Holmes, Geo. P. Ketchum, F. C. Eddelman, N. B. Stump, R. A. Alloway.

Each of whom had sustained a rigid examination. After the degrees were confered and the bibles presented—one to each member of the class—Prof. Spalding delivered a very excellent address, (which we shall present in the pages of the Register) which was responded to most beautifully, and impressively by Dr. W. H. Pabodie on behalf of the graduating class. The exercises were interspersed with most soul stiring music by Menter's celebrated Band. Thus closed the most interesting session, with the largest class, ever held in this institution.

